# Internet of Things: Development Intelligent Programmable IoT Controller for Emerging Industry Applications

**DOI:** 10.3390/s22145138

**Published:** 2022-07-08

**Authors:** Ti-An Chen, Shu-Chuan Chen, William Tang, Bo-Tsang Chen

**Affiliations:** 1 Department of Business Administration, JinWen University of Science & Technology, New Taipei City 231307, Taiwan; nhchen59@just.edu.tw; 2 Department of Industrial Engineering and Management, National Taipei University of Technology, Taipei 10608, Taiwan; 3 Tsing Hua Youzhu Technology Services Co., Ltd., Taipei 10455, Taiwan; 4 College of Management, National Taipei University of Technology, Taipei 10608, Taiwan; t107749008@ntut.edu.tw; 5 Suncre Smart Auto Technology Co., Ltd., Tainan 70815, Taiwan; one@scsa.com.tw

**Keywords:** sensor information, Internet of Things (IoT), programmable logic controller (PLC), smart manufacturing, Theory of Constraints (TOC)

## Abstract

The Internet of Things (IoT) has become critical to the implementation of Industry 4.0. The successful operation of smart manufacturing depends on the ability to connect everything together. In this research, we applied the TOC (Theory of Constraints) to develop a wireless Wi-Fi intelligent programmable IoT controller that can be connected to and easily control PLCs. By applying the TOC-focused thinking steps to break through their original limitations, the development process guides the user to use the powerful and simple flow language process control syntax to efficiently connect to PLCs and realize the full range of IoT applications. Finally, this research uses oil–water mixer equipment as the target of continuous improvement and verification. The verification results meet the requirements of the default function. The IoT controller developed in this research uses a marine boiler to illustrate the application. The successful development of flow control language by TOC in this research will enable academic research on PLC-derivative applications. The results of this research will help more SMEs to move into smart manufacturing and the new realm of Industry 4.0.

## 1. Introduction

With the wave of global 5G and Industry 4.0, Internet of Things (IoT) technology is also widely used in industry. The concept of the fourth industrial revolution assumes the convergence of human and digitally controlled machines with the Internet and information technology [1]. Industry 4.0 has also led to the sublimation of smart manufacturing, which is accompanied by the digital transformation of enterprises to continuously improve the level of IoT information and communication software and hardware. IoT is one of the emerging technologies, and it certainly attracts the attention of many researchers [2]. Ever since the term IoT was first introduced in 1999 [3], it has been regarded as a fast-developing technology in the era of wireless communication and as a leading technology for Industry 4.0 [4]. With the rapid development of the IoT worldwide, there will be about 50 billion smart devices in operation in various industries by 2025 [5,6], which shows the huge business opportunities of the IoT.

The importance and quantity of electronic products are increasing day by day [7]. IT technologies have contributed to the booming development of Industry 4.0 [8]. Born in the 1960s at General Motors in the US, programmable logic controllers (PLC) are used to solve the problems of time-consuming circuit modification of the electrical sequence control system, time management, and machine maintenance. PLCs have been applied for more than 50 years, but their prices and costs remain high, out of reach of most small and medium enterprises (SMEs).

A preliminary study reported conducting an experiment to identify bottlenecks in the software development process (SDP) [9]. Another study [10] showed where the Theory of Constraints (TOC) could be applied in software development, but did not show how the TOC could be used for this purpose. Ribeiro et al. [11] used the TOC in the software development process. They suggested that subsequent research could use the theory of constraints to explore bottlenecks in the software development process and make relevant process adjustments. The TOC aims to address bottleneck management so that overall operations can be improved and the maximum benefits can be achieved [12]. However, since constraints are any values that can prevent a system from achieving its goals [13], we must use the TOC to remove these limitations, especially in new product development (NPD) [14].

The purpose of this study is to develop a set of controllers with integrated wireless transmission and PLC control functions by applying the TOC and to explore the following five features for PLC applications to enable users to control PLCs more easily. The five main features include the following: (1) Process control syntax should be simple: develop a set of process control syntax, through simple instructions can be process control, process control. (2) The wireless transmission function is required: develop a set of IoT controllers with built-in DI (Digital Input)/DO (Digital Output), RS-232, and Wi-Fi connections. (3) User-friendly Software as a Service (SaaS) platform: establish a web browsing web platform to enable users to see the PLC on the web. (4) Social Software Push: the controller can message using the messaging app Line throughout the process to inform users of relevant information in real time. Finally, (5) real-time monitoring function: mobile devices can be used to monitor the status of the relevant IO in real time.

This research uses an oil–water mixer as a case study. In this research, the original relay and timer were removed, and an intelligent programmable controller developed in this research was installed to monitor the changes in water volume in real time and to recognize abnormal phenomena by automatically generating a Line push signal. In addition, this research applies restriction theory in R&D practice, hoping to make a real contribution to the expansion of restriction-theory-related applications. Finally, we hope to enable more small and medium-sized enterprises to invest in IoT deployment to promote the bright future of Industry 4.0.

## 2. Literature Review

### 2.1. Smart Manufacturing

According *The Global Lighthouse Network Playbook for Responsible Industry Transformation 2022* white paper [15] from the WEF (World Economic Forum), the Global Lighthouse Network has grown from 16 to 103 sites since 2018. The “Lighthouse Factory” competition is a collaboration between the WEF and McKinsey & Company and aims to select smart factories that have demonstrated outstanding results in the integration of cutting-edge technology applications in the field of Industry 4.0, including the use of automation, the industrial Internet of Things (IIoT), AI (Artificial Intelligence), AIoT (AI and IoT), digitization, big data analytics, 5G, etc. Smart Factories with excellent technological performance are invited to compete. In the above report, six core enablers are identified as key to a successful Fourth Industrial Revolution transformation: an agile approach, an agile digital studio, an IIoT stack, an IIoT academy, the technology ecosystem, and the transformation office.

Research shows that the main components of Industry 4.0 are the integration of system components and the digitization of manufacturing/service operations [16,17,18,19]. The six pillars that support companies in advancing smart manufacturing include (1) manufacturing technologies and processes; (2) materials; (3) data; (4) predictive engineering; (5) achievability and resource sharing; and (6) networks. However, these six pillars exist as nomenclature explanations in the enterprise [20]. The essence of manufacturing remains the same: it must offer low-cost, high-quality, and short-delivery manufacturing capabilities to provide maximum value to customers, obtain orders, and improve the sustainable competitiveness of the company [21].

Enterprises can use Industry 4.0-related technologies to integrate software and hardware in production control in order to automate the replenishment, manufacturing, monitoring, adjustment, and timely improvement of production processes in an Industry 4.0 production environment, thus significantly improving the efficiency of business operations [22]. The concept of the fourth industrial revolution assumes the integration of people and digitally controlled machines with the Internet and information technology [1]. The full digitalization of production processes is a very important part of the Industry 4.0 concept to improve the efficiency of manufacturing in small- and medium-sized enterprises (SMEs). Appropriate technologies must be used for rapid digitization, data transfer [23], data storage, and, ultimately, for data mining [24].

Industry 4.0 will evolve the industrial manufacturing process and product quality optimization from the previous judgment based on experience and observation to a complete intelligent system based on facts (data and events), which analyzes data to explore potential values [25]. For example, the application of the cloud platform can help enterprises to realize unified data management and support fast data retrieval and information sharing for different customer groups [26]. The cloud platform can eliminate the problem of Enterprise Resource Planning (ERP), Manufacturing Execution System (MES), and Supply Chain Management (SCM) becoming information silos and can also manage information from multiple sources simultaneously [27,28].

### 2.2. IoT

**Figure 1:**
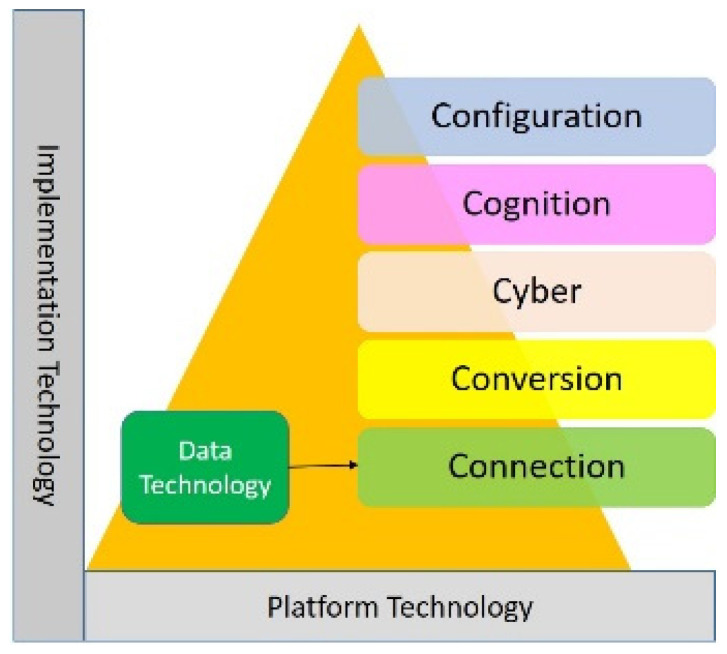
Location of intelligent sensing layer in the CPS manufacturing domain.

The IoT enables a wide range of devices to communicate and alternate information with each other, enabling a wide range of industry sectors to be enhanced [29]. Smart factory equipment requires the installation of intelligent sensors to collect a large amount of data related to the production process, making data critical in increasing the competitiveness of the industry [30]. Professor Lee Jay proposed a systematic approach called “Industrial Artificial Intelligence” to implement artificial intelligence [31,32,33,34], the 5Cs framework of this Cyber Physical System (CPS). The 5Cs of this CPS are Connection, Conversion, Cyber, Cognition, and Configuration (Figure 1). The first task at the machine level is to ensure the quality and comprehensiveness of the data from the source and the acquisition and management methods to build the foundation of the data environment at the upper level of the CPS. In addition to the structural environment and acquisition channels, another core concept is to automatically select the appropriate data acquisition method according to the goals and analysis requirements [25].

PLC controllers are one of the basic elements of the intelligent sensing layer. PLCs are electronic devices with built-in microprocessors whose main function is to control the operation of machines and other technical devices. The PLC input system collects information about the status of the device and the engineer’s operating instructions, and its output system has the main function of setting up connected actuators and signaling components and handling data transmission [1].

Using the Internet of Things and Cyber-Physical Systems (CPS), items such as materials, sensors, machines, products, supply chains, and customers can be interconnected. Each machine can independently and autonomously exchange information and control its own behavior during the production process, making it possible for the product to control its own production process [35].

The importance of IoT-related technology development and application is increasing day by day [36,37,38,39]. The collection of data on industrial implementation processes in the industrial environment is a critical aspect of the IoT [40]. Given the complexity of industrial operations, it is often difficult and costly to accurately collect data on the status of operations [41,42]. The approach proposed by Zhang et al. [43] is useful for decentralized learning of fault diagnostic problems in the context of data harvesting by different end-users while taking into account the security of their data. A generic domain adaptation method for fault diagnosis was proposed in [44] and experimentally validated on two rotating machine datasets, which is promising for practical applications under data uncertainty. The IoT is used in a wide range of applications, such as the application of large-scale sensing infrastructure to air quality monitoring systems. The solution proposed in [45] meets the requirements for real-time and large-scale processing of applications.

The IoT and Smart Manufacturing form the basic core of Industry 4.0. During production, machines and associated inspection components can collect and share production data in real time as the end result of the production process. Production quality can provide insight into machine status through backward reasoning algorithms. It can also provide feedback for system management to adjust production planning [46]. IEC 61131 is the international standard for programmable logic controllers (PLCs). The third part of the standard concentrates on the basic software architecture with programming language [47]. The instruction table (IL) language occupies a special place among programming languages. It is an assembly-like language in which all other program representations, graphical and textual, can be converted to IL [48]. PLC-based systems remain necessary and essential in most solutions [49,50].

The data collected by the PLC are quite useful, for example. Machines require preventive maintenance through data collection and analysis of parameters in addition to regular maintenance. It is prudent to be warned of machine failures before they occur, thus reducing the occurrence of unplanned downtime [51,52].

### 2.3. Theory of Constraints

The Theory of Constraints (TOC) was first advocated in production management by Goldratt and Cox (1984) in their book *Goals*. The TOC was introduced in the mid-1980s [53,54] and has had a significant impact on productivity improvement in manufacturing systems [55]. It has had great theoretical and practical diffusion in industry [56], and its activities have improved reliability [57]. A constraint, or bottleneck, is anything that limits the system from achieving higher performance [58]. Therefore, it is critical to identify bottlenecks and manage the organization by identifying and addressing bottlenecks since reductions in bottlenecks lead to improvements in the overall system [56].

The concept of the TOC can be summarized as [59]: (i) every system must have at least one constraint, and (ii) the presence of a constraint represents an opportunity for improvement.

The working principle of the TOC consists of five focusing steps:

(1) Identify the system’s constraint(s).
(2) Decide on how to exploit the system’s constraint(s).
(3) Subordinate everything else to the above decision.
(4) Elevate the system’s constraint(s).
(5) Warning! If in the previous steps a constraint has been broken, go back to Step 1, but do not allow inertia to create a system constraint [59,60,61].

Among the five steps of the TOC’s application, previous studies have differed on the importance of each step. Steps (1) and (4) are key to the successive application of the TOC methodology in companies [62]. In another study [61], the unit of analysis to be investigated is a single case study of a company in which the implementation of the TOC was analyzed, focusing on the third step of the methodology.

For researchers, constraints are relevant to problem solving [63]. Ikeziri et al. [64] conducted a bibliometric analysis of TOC research, sampling 1009 journal articles published from 1984 to 2019. That study divides the body of knowledge of the TOC into six knowledge domains that have been explored in the literature: (1) production; (2) projects; (3) yield accounting (TA); (4) thinking process (TP); (5) supply chain management (SCM), including applications in distribution and retailing; and (6) continuous process of improvement.

Although the TOC originated in industrial practice, it is believed that the concept is also applicable to other areas, such as service organizations [65,66]. Since its introduction, the TOC has been highly praised, and most companies have tried to adopt this approach to improve their competitiveness [55,65,66]. The TOC can reconcile risk factors and project planning organization and can reduce uncertainty in project construction schedules more effectively [67]. Recently, the TOC has been discussed as a lens to identify production and market bottlenecks in electric vehicle propagation as a strategy for companies [68]. Ribeiro et al. [11] investigate academic studies on the application of the TOC in software development processes. They also searched for its application in other production environments, trying to visualize methods that can be adapted to software development processes, such as studies on process optimization, process improvement, and process scheduling.

## 3. Materials and Methods

In this research, an Intelligent Programmable IoT Control was developed using the five stages of TOC. The first two stages were analyzed and then executed, following the TOC’s usage guidelines. In order to identify possible bottlenecks in the development process, this research concluded from the brainstorming of the team members that the bottleneck of this development project lies in the use of the controller program. This is because the current controller software must be operated by professionals, creating powerful barriers to its wide use by SMEs in the future.

In the second step, we consider how to reduce these barriers to use. Therefore, the design of the HMI human–machine interface promotes ease of use. We also investigate how to make the controller’s execution software “modular” and “structured”. In addition, since many manufacturing user environments do not have physical network lines, enabling wireless transmission for the controller is also one of the factors that affects willingness to use the system.

In the third step, this research addresses the limitations of the first two steps and applies countermeasures. The development team established an implementation plan. When all the countermeasures followed the above decision, this research then developed improvement countermeasures that were able to efficiently link PLC to achieve the full range of applications of the Internet of Things.

**Figure 2:**
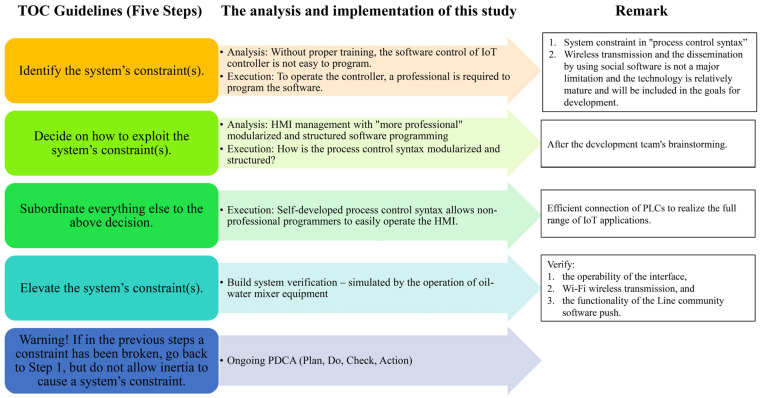
Analysis and implementation of the five phases of the TOC in this research.

In the fourth step, we validated the countermeasures proposed in this research by simulating the operation of the oil–water mixer equipment to verify (1) the operability of the operating interface; (2) Wi-Fi wireless transmission; and (3) the function of the Line community software push. Step 5 did not occur in this research. If the previous steps had broken through the constraints, we would have returned to step 1 and not let the inertia cause a constraint in the system, and we would have used the PDCA (Plan, Do, Check, Action) cycle to implement this step. The implementation of the five stages of the TOC in this research is shown in Figure 2. This chapter will explain the development of new product functions, using Suncre Flow Language.

### 3.1. Development of New Product Functions

NPD refers to the innovation process of production technology, the core task of which is to develop appropriate system solutions for innovative ideas [69,70,71]. We used one controller with Web integration + PLC control. This Wi-Fi controller is built into the control module and can be used to check the current status via the Web as long as it is connected to the Internet. It may also be used to receive messages via Bluetooth from a mobile device, even locally. It has two major features:

1 Combining the functions of the IoT and a PLC programmable controller, the PLC programmable controller can be connected to the network without the need to install devices other than the controller to realize off-site monitoring and automatic return of abnormality information.
2 The previous PLC + Human–Machine Interface (HMI) design can only view the relevant information at the local end. If the user wants to view the relevant IO status or status at the remote end, the user must add another work computer and additional commissioning costs to check the IOs and their status on the offsite side.
3 Hardware specifications:
  ● 8ea 24V digital input points;
  ● 4ea 24V digital output points;
  ● Two sets of simple RS-232 serial transmission (RX/TX/GND);
  ● Built-in wireless network communication function.
4 Software Specifications:
  ● In addition to hardware-related IO and Wi-Fi control, the following specifications are included:
  ● Provide 30 sets of dedicated control syntax, such as setting output, capturing input, etc.;
  ● 10 groups of Count functions and 10 groups of Timers;
  ● Provides 100 virtual integer memory locations;
  ● Provide macro (subroutine) commands for easy programming.
5 External communication function:
  ● The built-in command can be combined with Line push to send an active message to inform users of abnormalities;
  ● The built-in command can be used to send signals to the Server via Wi-Fi;
  ● RS-232 communication function can be carried out by built-in command;
  ● Based on the above, the major functions of the intelligent programmable controller developed in this research are shown in Table 1.

### 3.2. Flow Language

**Figure 3:**
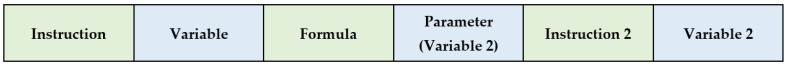
Syntax instruction structure diagram.

The control syntax of this design adopts Intuitive Programming Language (SunCre Flow Language, SCFL for short in this study), which is a set of self-developed flow control syntax and a set of simple control flow syntax, which can import flow information into the controller and control-related IO actions through text files only. This study stores pre-defined functional programs in a modular form in the coding system, eliminating the need to repeat all the contents of the modular programming language when writing in the progressive language. The industrial nature is repetitive, mass-produced, and standardized, so the application of individual programming language to industry is very suitable. The basic structure of process control is shown in Figure 3.

### 3.3. Intelligent Programmable Controller Development Focus

The intelligent programmable controller developed in this research focuses on five major items of software design: (1) language flow framework development; (2) PC-based tool design; (3) Web-based architecture development; (4) Web-based architecture development; and (5) controller firmware programming design. The project-design-related content description is presented in Table 2.

## 4. Results and Discussion

**Figure 4:**
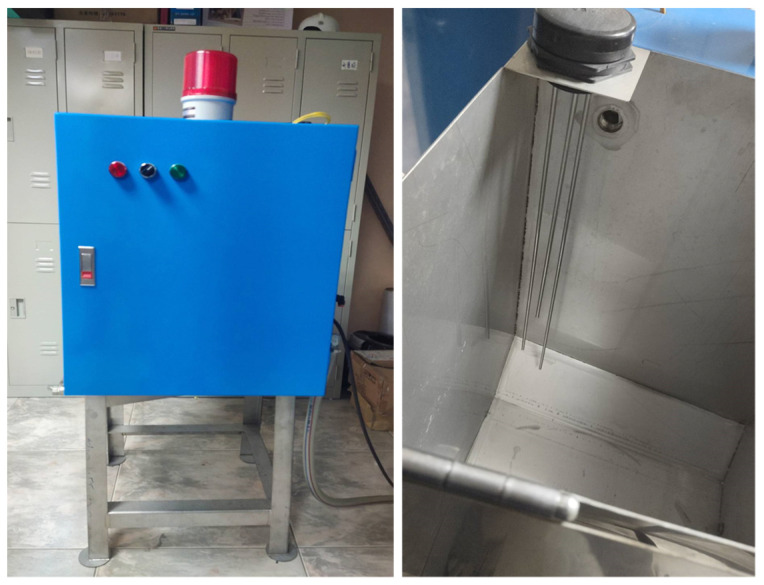
Oil–water mixing machine equipment.

In this research experiment, an oil–water mixer is used as a case study. The structure of the mixer barrel is shown in Figure 3. If the water level is lower than requested, water is added, and the water source is turned off after the water reaches the correct level. If there is an abnormality, it will trigger an abnormality signal. The control panel after opening the protection door is shown in Figure 4. Figure 4 shows the appearance of the oil-water mixer and the position of the internal sensors. The three lights on the left are, from right to left, the power light, the start switch, and the abnormal light. The other three lights on the right, from right to left, are the common sensing point, full water level sensing, and low water level sensing.

**Figure 5:**
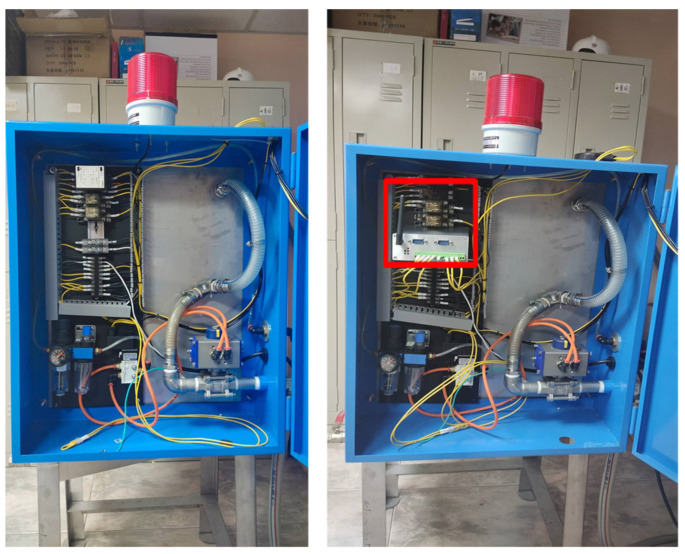
Oil–water mixer with the Intelligent Programmable IoT Controller.

The modified oil–water mixer equipment is shown in Figure 5. The right figure in Figure 5 represents the installation of the Intelligent Programmable IoT Controller developed in this study, the left figure in Figure 5 is before installation, and the red box indicates the installation location of the Intelligent Programmable IoT Controller.

### 4.1. Controller Planning

For the oil-water mixer, a total of five input signals and three output signals are used in this research. The Bool and Timer registers are also used. The plan is shown Table 3. The input signals are either sensor signals or pushbuttons, with five settings from X_0_ to X_4_. The output signals are mainly for the control of relays or pneumatic cylinders and are set from Y_0_ to Y_2_. In addition, the input and output signals are independent and do not correspond to each other.

The Register Planning of the controller is given in Table 4. The Bit column represents the digital data registers of the 13 systems from B_0_ to B_12_; Count is the system’s count register, and currently, only one C_1_—Water supply Time Out—is used. The two systems are independent and have no corresponding relationship. The blank column shown in Table 4 is because these functions are not used and therefore appear blank.

### 4.2. Controller Planning

**Figure 6:**
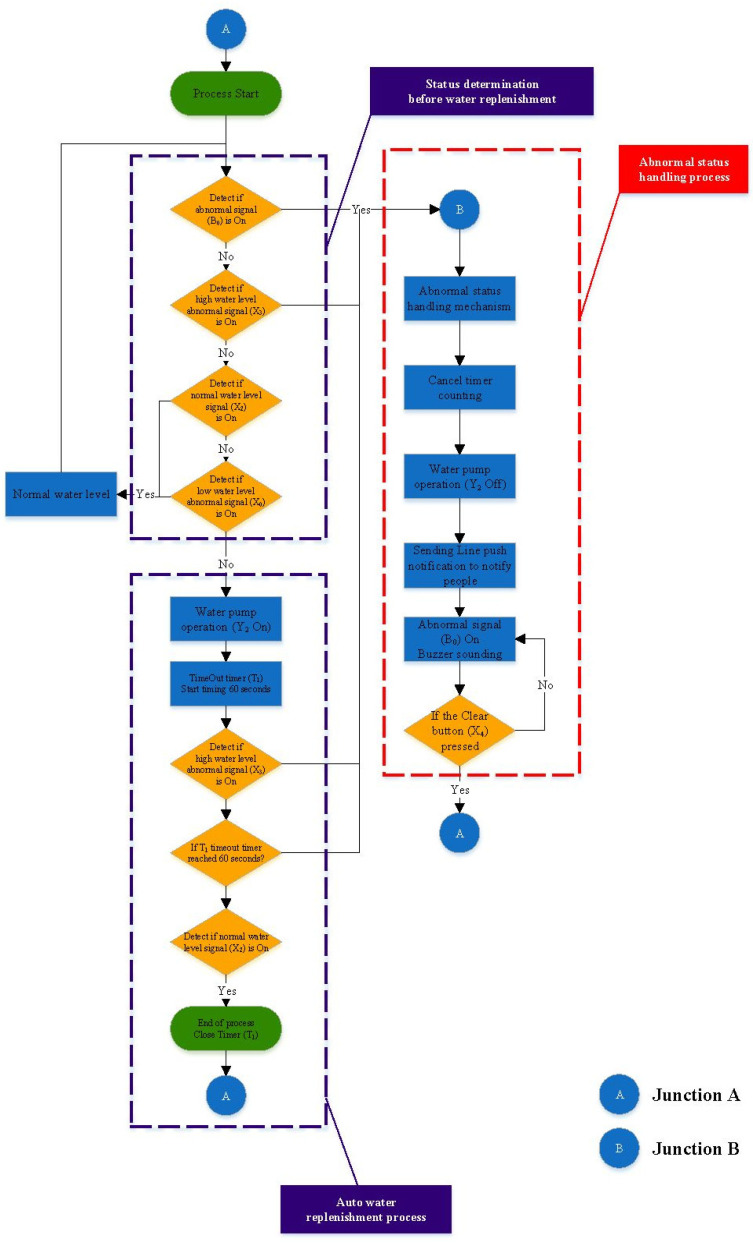
Controller planning flow chart.

In this research, the IoT Control of the oil–water mixer is used as the object for verification. The development of the controller process comprises three modules: (1) state determination before automatic water filling; (2) automatic water filling control process; and (3) the abnormality handling mechanism. The controller planning process is shown in Figure 6. The three major control programs in Figure 6 (Status determination before water replenishment, Auto water replenishment process, Abnormal status handling process) are the (1) initialization program, (2) operation program, and (3) control program.

### 4.3. Controller Process Control Program Instructions

Figure 6 displays the controller planning flow chart. Based on that flow chart, we wrote the process control program instructions as follows:

1. Main program:

Read the status of the four input points in a loop. If the status meets the settings, then proceed to the subsequent related actions.

Each step that should be executed in the process is written as a subroutine, and the subroutine process will be carried out only when the relevant settings are met.

2. The subroutines are divided into three main parts:
  (1) Executing the process;
  (2) Start Timer to prevent Sensor abnormalities such as water overflowing from the bucket of water if added to the bucket for too long;
  (3) Line pushing when an abnormality is detected.

**Figure 7:**
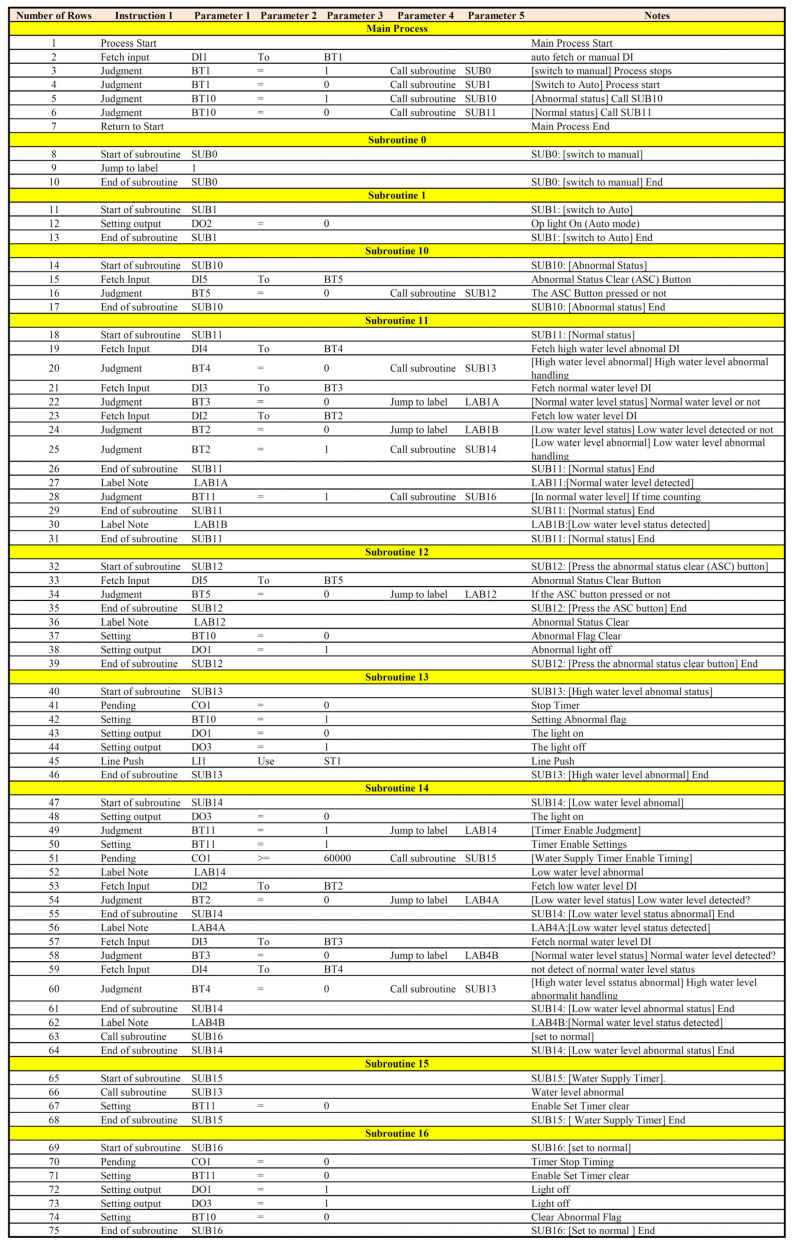
Process control program instructions.

There is only one main program for each control process, but there can be many sub-programs. The main program starts with “Process Start” and ends with “Return to Start”. When the program runs to return to start, it will return to the first line of the program and start again. According to Figure 6, the controller planning flow chart, this research writes the process control program instructions as shown in Figure 7 below. The process of Figure 6 may be compared with the relevant process control program instructions of Figure 7.

**Figure 8:**
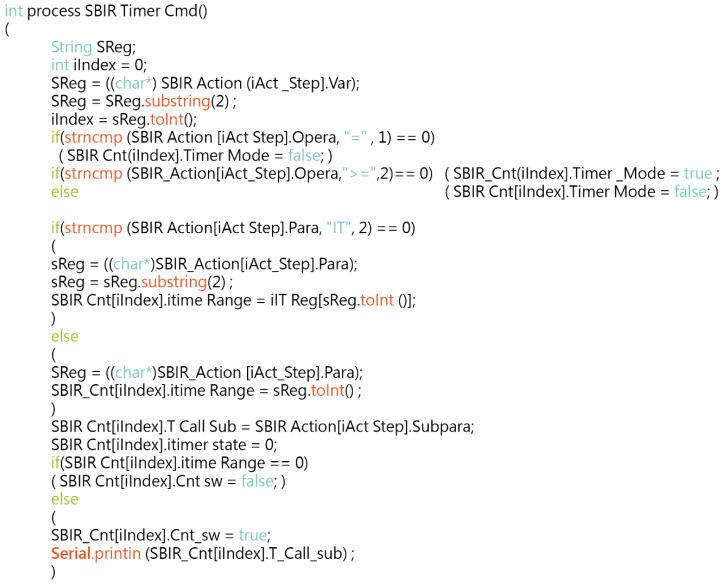
The source code of the timer function.

Take the check timer function as an example, the program source code in this section is related to the time timer element (C_0_) used in the system. In this study, an intuitive programming language was used to program the timer initialization, runtime, and control programs into five modules, namely: (1) obtaining the timer counter to be set; (2) confirming the type of judgement—the operation formula; (3) obtaining the time to set the Timer; (4) the Sub to be called at timeout; and (5) starting or stopping the timer counter. The function can operate against time variables, such as waiting for 5 s and then continuing to the next action. the source code of the timer function is shown in Figure 8.

### 4.4. Web Screen Display on the User Side

**Figure 9:**
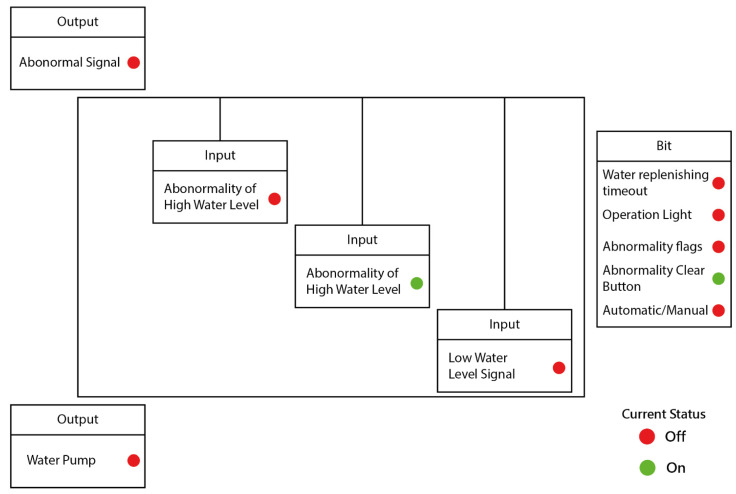
User-end web screen.

The web screen is displayed in Figure 9. By setting the protocol to send data to the Server every 30 s, the information of the controller is displayed on the screen immediately, enabling the personnel to check the current status of the controller through the web.

The image in the screen (Figure 9.) is the template of the background image. In addition, the data display of each IO status can be dragged to a fixed location and stored by the designer as desired. Part of the Web screen can be displayed through an intuitive system. Each control point can display not only the XY signal but also the internal register information such as the Bit register information, the Internal register, and the Count information. The lights in Figure 9 illustrate that status of the light is green when the sensor is on and red when it is off.

### 4.5. Mobile Device (Cell Phone) Screen Display

**Figure 10:**
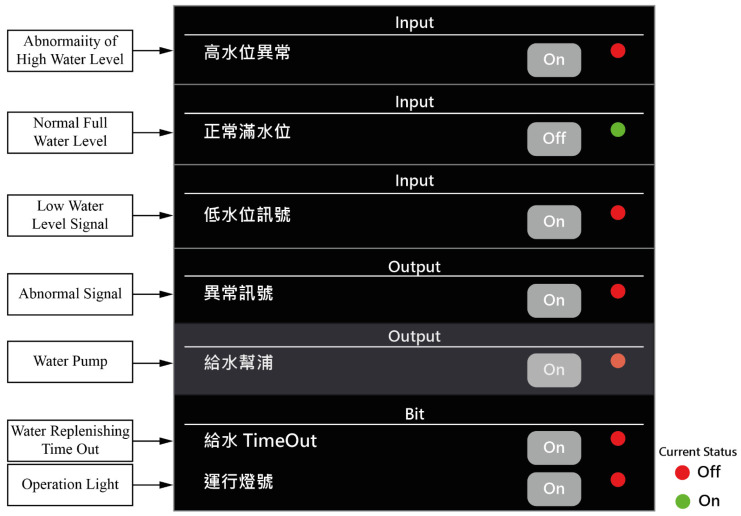
Mobile device (cell phone) screen display—part 1.

**Figure 11:**
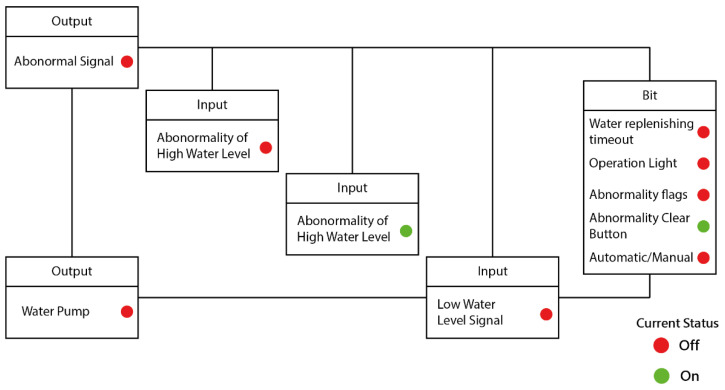
Mobile device (cell phone) screen display—part 2.

The same information is displayed on the phone as shown on the screen below (Figure 10 and Figure 11). The screen shows the IO status and the real-time display information with a background image. The lights in Figure 10 and Figure 11 illustrate that the status of the light is green when the sensor is on and red when it is off.

### 4.6. Abnormality Message Line Push Broadcast Function

**Figure 12:**
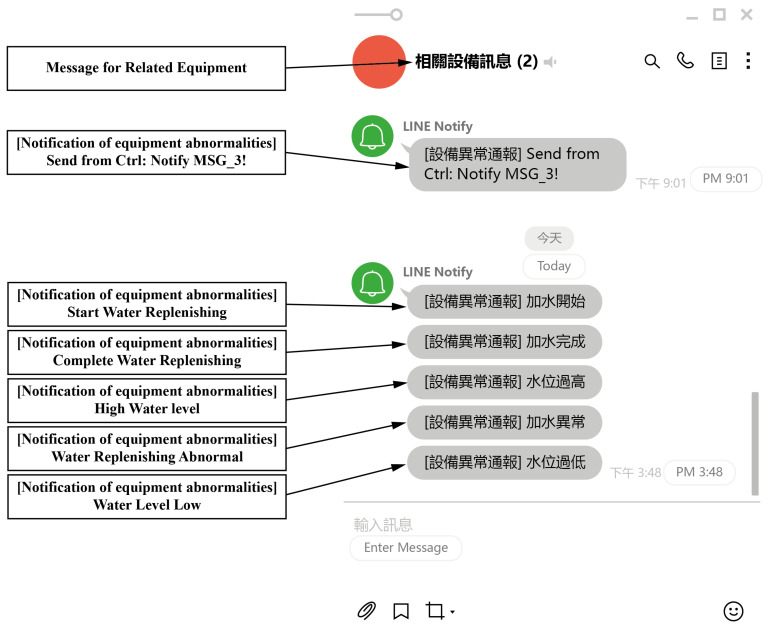
A screenshot of an abnormality message Line Push broadcast function.

In the control flow, the user can push the preset commands to the phone by the Line command “Line Push”. The actual screen is shown below (Figure 12), and the related text information can be set through the setup screen.

### 4.7. Discussion

For a small control system (eight input signals, four output signals), the previous PLC and HMI design can only display the information locally. To minimize the cost of the existing IoT devices is one of the main challenges [72]. If the user wants to view the system status in a different location, another computer must be added to view the system status from a different location, and the programming and related hardware are costly and require professional design help. Furthermore, a person must visit the site to make the relevant program modifications, raising costs and processing time. This controller integrates the functions of web browsing and serial connection and control of PLCs with one machine. There are three main features: (1) Off-site monitoring through mobile devices: Combining the functional features of IoT and PLC, this controller can mainly realize off-site monitoring and real-time automatic exception information. (2) No need for additional work computers and external programming: Traditional PLC and HMI design can only browse information at local machine, if the user wants to check the IO or other related status of the equipment at remote side, who needs to invest in additional hardware and software development. (3) The intuitive programming language (SunCre Flow Language, SCFL) developed in this research allows engineers to learn and use it in a short period of time.

### 4.8. Application for IoT Controller in the Marine Industry

The boiler is an important piece of equipment in a vessel. The steam generated by the boiler is used to heat fuel oil or cargo oil and to drive the boiler for cargo oil pumps, boiler feed pumps, and daily miscellaneous use. Auxiliary boilers consist of fuel oil boilers and exhaust gas boilers. They mainly use the heat energy from the fuel oil or diesel engine to produce steam (or hot water). When a boiler is equipped with a PLC, the PLC can monitor (1) the water supply pump, (2) the water supply temperature, (3) the furnace temperature, (4) the feedback water temperature, (5) the burner, (6) boiler water replenishment, (7) the furnace water level, and (8) the furnace pressure of the marine boiler in a timely manner.

**Figure 13:**
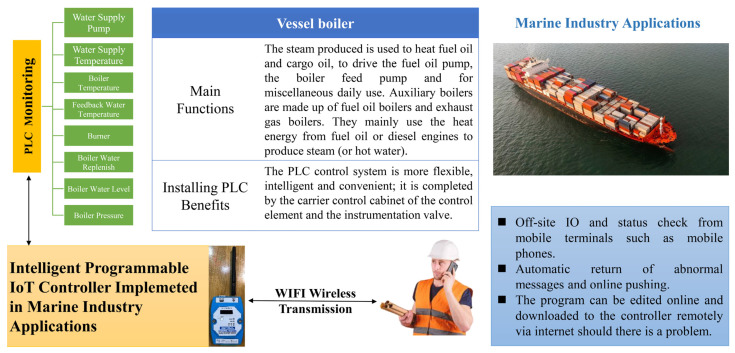
IoT Application Scenario for Vessel Boiler in Marine Industry.

Before the IoT controller developed in this research was installed, a dedicated person was required to control the boiler at the control station. However, the installation of an IoT controller with simple control and wireless transmission, which has been tested in this research, will enable real-time monitoring and wireless transmission of reports of work or abnormalities, which will bring the following three benefits: (1) the IO and status can be checked from a mobile terminal such as a mobile phone, (2) abnormal messages can be sent back automatically and can be pushed online, and (3) the program can be edited online, so that if there is a problem with the program, it can be downloaded to the controller via the Internet. The scenario of an IoT application for a vessel boiler in the marine industry is shown in Figure 13.

## 5. Conclusions and Recommendations

In this era of intelligent systems, IoT data collection remains an unattainable dream for non-IT professionals, especially in small- and medium-sized enterprises (SMEs), where resources are scarce. Thus, streamlining the process of moving towards smart manufacturing is critical for smaller firms. This research designs an IoT solution that does not require a computer hardware device but can design the control process directly on the mobile device and can display its signal changes on a webpage. The design goal is to provide convenient design, intuitive status, and timely notification of abnormality status through integration with the messaging APP Line, enabling companies to invest in smart manufacturing with minimal resources.

### 5.1. Conclusions

The traditional use of the controller requires professional IT personnel and engineers to work together. However, the intelligent programmable controller developed by this research can be set up and operated by a single engineer, which makes it suitable for small and medium-sized enterprises to implement Industry 4.0 concepts and integrate data collection with digital transformation. In addition, the future proposed price of the controller will be lower than that of existing controllers in the market, which will increase the willingness of enterprises to purchase and use the controller.

This research uses the Theory of Constraint to identify the bottlenecks of current PLC controllers, and then addresses these limitations to develop improvement measures to remove them. These measures include (1) modular HMI operation; (2) wireless transmission; and (3) real-time messaging via Line. The Intelligent Programmable IoT Controller is easily operable and can be applied to the manufacturing sites of SMEs since information can be wirelessly transmitted without restriction.

### 5.2. Research Limitations and Suggestions for Future Research

This research is a practical application research, not a theoretical research or an application of a new model. It is hoped that this research can be used to develop a more advanced manufacturing process or implementation of Industry 4.0 for a wider range of SMEs. Since the validation scenario in this research is a wireless transmission environment without external interference, the validation was not conducted under the interference of a real wireless communication environment; this is one of the limitations of this research. This research suggests that future research should be conducted in a more demanding wireless environment. It is also recommended that future research should explore the network security of PLC controllers to enable a wider range of SMEs to implement smart manufacturing more smoothly and cost-effectively in secure wireless transmission environments.

## Figures and Tables

**Table 1:** Controller System Functions.

Main Features	Contents
Hardware Information	8ea DI (Digital Input)/4ea DO (Digital Output)
Programmable	Yes
Communication Connectivity	Bluetooth/Wi-Fi/RS232
Line Push	Yes
IoT remote monitoring	Yes
HMI function	Yes
Mobile Bluetooth connection	Yes

**Table 2:** Software Design Project Description.

Development and Design Focus	Development and Design Content
SCFL Framework Development	(1) DIO read/set command design. (2) Register Bit/Word read/set instruction. (3) Counter function instruction. (4) Subroutine function instruction. (5) Judgment function instruction.
PC-based tool design	The PC Utility has three main functions: (1) Setting through RS-232 connection: ● IP information required for Internet access. ● Line Push Token. ● IO name. ● Register variable name. ● Exception message. (2) Programming syntax. Commands can be edited on a PC and downloaded to the controller. (3) Easy man-machine interfacing. A set of simple man-machine can be monitored after the basic base map is loaded and the object is created. When there is an abnormality for personnel to check, the information will be displayed on the right side of the screen.
Web-based architecture development	(1) Backend database development equipment data, customer data, related database design, cloud system setup. (2) Front-end web page information display. User (customer) login screen design. Controller information display status screen design.
Mobile device tool programming	(1) Complete cell phone APP function: users can edit the desired control process via mobile phone. (2) It can transmit commands to the controller via Bluetooth. (3) A man–machine screen for mobile phones is constructed, for convenient notification of the location of abnormality signals. (4) Parameter setting function design.
Controller firmware programming design	(1) Complete internal firmware design of the controller to enable the controller to monitor processes following input commands. (2) Complete external communication functionality for the controller, including Wi-Fi, Bluetooth, and RS232 wired monitoring function. (3) The controller can directly connect to Web and push messages through Line. (4) SCFL universal command design. (5) SCFL subroutine and interrupt function flow design.

**Table 3:** Input and Output Signal Planning.

	Input Signal		Output Signal
X_0_	Automatic/Manual	Y_0_	Abnormal light signal
X_1_	Low water level signal	Y_1_	Functioning light
X_2_	Normal full water level	Y_2_	Water pump
X_3_	Abnormal high water level		-
X_4_	Abnormality Clear Button		-

**Table 4:** Register Planning.

	Bit		Output
B_0_	-	C_01_	Water supply Time Out
B_1_	D1-1 Push button use		-
B_2_	D1-2 Push button use		-
B_3_	D1-3 Push button use		-
B_4_	D1-4 Push button use		-
B_5_	D1-5 Push button use		-
B_6_	-		-
B_7_	-		-
B_8_	-		-
B_9_	-		-
B_10_			-
B_11_			-
B_12_	-		-

## Data Availability

The data are not publicly available due to its use on future research activities.
